# The relationship between time to diagnose and diagnostic accuracy among internal medicine residents: a randomized experiment

**DOI:** 10.1186/s12909-021-02671-2

**Published:** 2021-04-21

**Authors:** J. Staal, J. Alsma, S. Mamede, A. P. J. Olson, G. Prins-van Gilst, S. E. Geerlings, M. Plesac, M. A. Sundberg, M. A. Frens, H. G. Schmidt, W. W. Van den Broek, L. Zwaan

**Affiliations:** 1 Erasmus Medical Center Rotterdam, Institute of Medical Education Research Rotterdam, Rotterdam, The Netherlands; 2 Department of Internal Medicine, Erasmus Medical Center Rotterdam, Rotterdam, The Netherlands; 3 Division of General Internal Medicine, University of Minnesota, Section of Hospital Medicine, Minneapolis, USA; 4 Department of Internal Medicine and Department of Infectious Diseases, Academic Medical Center Amsterdam, Amsterdam, The Netherlands; 5 Department of Neuroscience, Erasmus Medical Center Rotterdam, Rotterdam, The Netherlands

**Keywords:** Cognitive bias, Decision making, Diagnostic error, Patient safety

## Abstract

**Background:**

Diagnostic errors have been attributed to cognitive biases (reasoning shortcuts), which are thought to result from fast reasoning. Suggested solutions include slowing down the reasoning process. However, slower reasoning is not necessarily more accurate than faster reasoning. In this study, we studied the relationship between time to diagnose and diagnostic accuracy.

**Methods:**

We conducted a multi-center within-subjects experiment where we prospectively induced availability bias (using Mamede et al.’s methodology) in 117 internal medicine residents. Subsequently, residents diagnosed cases that resembled those bias cases but had another correct diagnosis. We determined whether residents were correct, incorrect due to bias (i.e. they provided the diagnosis induced by availability bias) or due to other causes (i.e. they provided another incorrect diagnosis) and compared time to diagnose.

**Results:**

We did not successfully induce bias: no significant effect of availability bias was found. Therefore, we compared correct diagnoses to all incorrect diagnoses. Residents reached correct diagnoses faster than incorrect diagnoses (115 s vs. 129 s, *p* < .001). Exploratory analyses of cases where bias was induced showed a trend of time to diagnose for bias diagnoses to be more similar to correct diagnoses (115 s vs 115 s, *p* = .971) than to other errors (115 s vs 136 s, *p* = .082).

**Conclusions:**

We showed that correct diagnoses were made faster than incorrect diagnoses, even within subjects. Errors due to availability bias may be different: exploratory analyses suggest a trend that biased cases were diagnosed faster than incorrect diagnoses. The hypothesis that fast reasoning leads to diagnostic errors should be revisited, but more research into the characteristics of cognitive biases is important because they may be different from other causes of diagnostic errors.

**Supplementary Information:**

The online version contains supplementary material available at 10.1186/s12909-021-02671-2.

## Background

Diagnostic errors are a serious patient safety concern that went largely unrecognized [1] until the National Academies of Sciences, Engineering, and Medicine (NASEM) published the report ‘Improving Diagnosis in Healthcare’ in 2015 [2]. Understanding the underlying causes of diagnostic errors is a crucial step towards reducing those errors. Research findings of a variety of studies [3–6] have led to the consensus that cognitive flaws are a major cause of diagnostic errors [7–12]. However, researchers disagree about the type of cognitive flaw that is the main cause [13, 14]. The discussion is centered around the question whether cognitive biases or other cognitive flaws, such as knowledge deficits, are the most common cause of error [13]. In the diagnostic error literature, a common explanation is that errors are caused by cognitive biases due to fast reasoning and that slowing down and taking more time can prevent these errors [8, 10, 15, 16]. Contributing to clarifying the influence of time taken to diagnosis on the likelihood of making mistakes is of the utmost importance in determining what strategies may be effective in decreasing diagnostic errors.

Diagnostic reasoning is frequently described by dual process theory (DPT), an influential theory on decision-making in the field of psychology [17, 18]. DPT describes that reasoning consists of two systems, called System 1 and System 2 [18]. System 1 relies on heuristics (mental shortcuts) and on fast and automatic reasoning. We are only conscious of the final product of System 1 reasoning and therefore it is called *non-analytical reasoning*. On the other hand, System 2 is slow, sequential, and allows for deliberate reasoning, although the system is limited by the capacity of our working memory. System 2 reasoning is regulated: we are conscious of both the process and the result, and therefore it is called *analytical reasoning* [16–19]. The separation of System 1 and System 2 is primarily relevant in theory, as non-analytic and analytic processes tend to blend together in practice.

The shortcuts in non-analytical reasoning can introduce cognitive biases (predispositions to think in a way that leads to systematic failures in judgement [17]). An example is availability bias, where people rely on examples that come to mind easily; e.g. clinicians are more likely to diagnose a patient with the same condition as in a recently seen patient [6]. Based on this rationale, non-analytical (and therefore, fast) reasoning is purported to be a major cause of bias-induced diagnostic errors [7, 12, 15, 16, 20, 21].

To prevent such errors, many interventions stimulate slower, more analytical reasoning. However, this idea is contradicted by the studies of Sherbino et al. [22] and Norman et al. [23], who showed that faster diagnoses were more often or just as often correct as slower diagnoses. This implies that fast (or faster) reasoning cannot be equated to faulty reasoning and actually may lead to excellent diagnostic performance. It has also been suggested to only slow down when necessary to make sure that correct diagnostic processes are not disrupted; however, it seems that clinicians often do not know when they would require extra time or help. This was shown in a study by Meyer et al. [24] where clinicians’ confidence and their intention to request for help (e.g. from a colleague) did not correctly reflect their diagnostic accuracy.

Despite these arguments, diagnostic errors are still primarily attributed to fast diagnostic reasoning [10, 15, 16, 20, 21] and the overall view of diagnostic errors has not shifted much. An important limitation of the studies showing that faster diagnoses were just as often correct as slower diagnoses is that they used a between subjects design and therefore can alternatively be explained by assuming that faster participants were just better diagnosticians than slower participants [22, 23]. Additionally, these studies only focused on correct versus incorrect diagnoses and did not examine how bias-induced diagnoses related to time to diagnose.

To determine how time to diagnose relates to diagnostic error within subjects, we induced availability bias (by using Mamede et al.’s methodological procedure for bias-induction [6]). First, residents evaluated the accuracy of simple cases and subsequently diagnosed a similar case with a different diagnosis. If they would provide the same diagnosis as they had evaluated before, this was considered an error due to availability bias. If they provided another incorrect answer, this was considered a diagnostic error due to other reasons. We compared their time to diagnose and confidence when they were correct, incorrect due to bias or incorrect for other reasons. Furthermore, we explored perceived case complexity and mental effort invested in diagnosis, and determined residents’ confidence-accuracy calibration and resource use to study how these measures would be affected by bias, the effect of which was not examined by Meyer et al. [24].

We expected to replicate Sherbino et al. [22] and Norman et al. [23]‘s findings, but now in a within-subjects design, and to show that faster reasoning was not necessarily related to diagnostic errors. Specifically, we expected that both bias-induced diagnostic errors and correct diagnoses would be diagnosed faster than other errors. Furthermore, we expected that confidence would be lower for both bias errors and other errors than for correct diagnoses.

## Methods

### Design

**Fig. 1:**
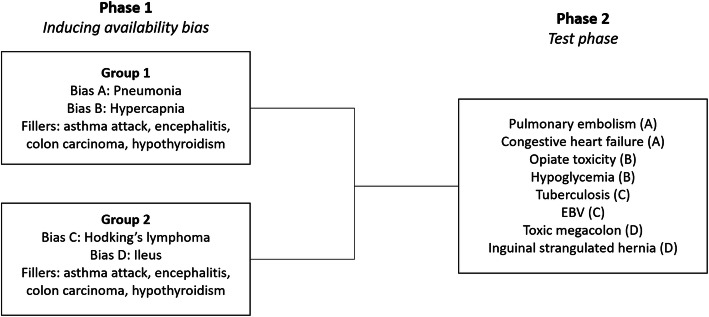
Study design and clinical cases shown in each phase

The study was a two-phase computer-based experiment with a within-subjects design (Fig. 1), based on a study by Mamede et al. [6] where availability bias was induced. All methods were carried out in accordance with the relevant guidelines and regulations. The experiment consisted of two phases, with no time-lag between the phases:

1) *Bias phase:* Residents were randomly divided into two groups, who each evaluated 6 clinical cases with a provisional diagnosis. Both groups saw four filler cases (cases meant to create a diverse case mix and to distract from the bias cases) and two biasing cases. The biasing cases were different for each group: residents in group 1 saw biasing cases A (pneumonia) and B (hypercapnia) and residents in group 2 saw biasing cases C (Hodgkin’s lymphoma) and D (ileus) (Fig. 1). This way, the two groups were biased towards different cases and acted as each other’s controls in the test phase. Additionally, creating two groups allowed us to correct for case complexity and increase generalizability.

2) *Test phase:* Residents diagnosed 8 clinical cases. Half of the cases were similar to the biasing cases shown to group 1; the other half were similar to the biasing cases shown to group 2 (Fig. 1). Thus, residents diagnosed four cases for which they saw the similar case in Phase 1 and four for which they did not, resulting in four cases that were exposed to bias and four cases that were not exposed to bias for each resident.

### Participants

In total, 117 Internal Medicine residents in their 1st to 6th year of training participated (Table 1). Group 1 and 2 consisted of 57 residents and 60 residents respectively. Residents were in training at one of the three participating academic medical centers: two in the Netherlands and one in the USA. Residents from the Dutch academic centers were recruited during their monthly educational day; residents from the American academic center were recruited individually (by APJO, MAS, and MP).

**Table 1:** Participant demographics

*Hospital*	*N*	*Age (SD)*	*N (%) Female*	*Years as resident (SD)*
Erasmus Medical Centre (Rotterdam, Netherlands)	26	31 (3.5)	14 (54%)	2.2 (1.1)
University Medical Center Amsterdam (Amsterdam, Netherlands)	69	35 (2.5)	47 (69%)	2.5 (1.3)
University of Minnesota (Minnesota, U.S.A.)	23	29 (2.0)	12 (52%)	1.6 (1.1)

Sample size was prospectively estimated in G-power [25]. We calculated sample size for an ANCOVA (analysis of covariance) with a medium effect size, a power of 80%, an α of 0.05, 2 groups and 2 covariates. This estimation indicated that 128 participants would be required.

### Materials

Sixteen written cases (Fig. 1) were developed by one internist and diagnosed and confirmed by another internist who was not aware of the diagnoses of the first internist (JA and GP). Cases consisted of a short history of a fictional patient, combined with test results (Additional file 1). Cases were designed in sets with the same presenting symptom, but each case had a different final diagnosis. Cases in each set were matched by superficial details such as patient gender and age. All cases were piloted (*N* = 10) to ensure appropriate level of difficulty. All materials were available in Dutch and English. An online questionnaire (Additional file 2) was prepared in Qualtrics (an online survey tool).

### Procedure

Residents received an information letter and were asked to sign informed consent. They were told that the goal of the study was to examine information processing during diagnosis when evaluating diagnoses, and when diagnosing cases themselves.

In the first phase (bias induction), residents estimated (on a scale from 0 to 100%) the likelihood that a provided provisional diagnosis was correct. All diagnoses were in fact correct. This was followed by a test phase in which residents were given 8 clinical cases for which they had to provide the most likely diagnosis as a free text response.

After diagnosing all cases, residents were shown the history of each case again and were then asked to provide for each case the confidence in their diagnosis, their perceived complexity, and their invested mental effort in diagnosing the case. We also measured residents’ confidence-accuracy calibration by correlating their average confidence and accuracy ratings. Lastly, we asked if they had wanted to use additional resources to diagnose the case.

Finally, we provided feedback by showing the cases, the diagnosis the resident had provided, and the correct diagnosis. For cases with a provisional diagnosis, we showed the residents’ indicated likelihood of the diagnosis being correct and told them that all provisional diagnoses had been correct.

### Outcome measures

The independent variable was the type of bias exposure: participants were biased to either cases A/B or to cases C/D (Fig. 1). The main dependent variable was the final diagnosis, which was defined as correct, bias error, or other error. A bias error occurred when the diagnosis from Phase 1 was given; other errors occurred when another incorrect diagnosis was given (other error). A diagnosis could only be defined as a bias error if residents saw the corresponding bias case in Phase 1 of the study; otherwise their diagnosis was labelled” other error”. Additionally, we calculated the frequency with which residents mentioned the bias diagnosis of a case in the control condition (when they did not see the bias case), which had to be significantly lower than in the bias condition. Otherwise, the ‘bias’ diagnosis could also be a probable differential diagnosis, which prevented us from concluding the error was made due to bias. This was scored by two internists (JA and GP), who independently assessed and assigned a score to all diagnoses. A score of 0 was given for incorrect diagnoses; a score of 0.5 was given for partially correct diagnoses (e.g. the participant answered sepsis, but the diagnosis was pneumonia with sepsis); a score of 1 was given for fully correct diagnoses. After the first ratings, their responses were compared and discrepancies were resolved through discussion.

We measured time to diagnose in seconds spent on each clinical case and confidence on a scale from 0 to 100%. We additionally measured case complexity [26] and mental effort [27, 28], also on a scale from 0 to 100%. The confidence-accuracy calibration was expressed by a goodness-of-fit (R^2^) measure through a scatterplot of average confidence and accuracy per resident. Finally, resource use was measured as the percentage of residents who wanted to use extra resources.

### Statistical analysis

First, we examined whether the bias induction was successful by comparing if the frequency with which residents mentioned the bias diagnosis in the control condition (when they did not see the bias case) was significantly lower than in the bias condition. This determined which comparisons we could analyze.

We then calculated the mean for time to diagnose, confidence, complexity, and mental effort over all cases for each error type. The time to diagnose variable was scaled prior to the calculation of the mean to correct for differences due to case length. This was done by calculating a grand mean from the individual means of all 8 cases and subtracting the grand mean from the individual means for time to diagnose. This indicated the number by which every individual time would have to be corrected and resulted in the scaled times to diagnose. Furthermore, per analysis we excluded residents for whom a mean could not be calculated due to missing values.

#### Statistical tests

We compared residents’ correct diagnoses, bias errors, and other errors on time to diagnose, confidence, complexity, mental effort, using two-sided repeated measures *t*-tests. The originally planned ANCOVA was not performed because we did not induce bias. We used three tests to compare these types of diagnoses instead of one encompassing test because such a test would unnecessarily exclude residents due to listwise exclusion. For each instance of multiple testing, the alpha level was corrected to α = .017 (.05/3) using a Bonferroni correction. Analyses were performed in Spyder (Python 3.7). Additionally, for each significant result we calculated the Cohen’s *d* [29] and the 95% confidence interval around the mean difference. The relation between resource use and diagnostic accuracy was evaluated using a repeated measures binomial logistic regression in Rstudio (version 1.2.5003), for which we calculated the odds ratio.

## Results

### Bias induction

A one-way analysis of variance (ANOVA) showed no significant difference in the frequency with which the bias diagnosis was given on cases that were exposed to bias and cases that were not exposed to bias (*p* > .05). Additionally, out of the 117 residents, residents infrequently mentioned the bias diagnosis (0–20 times for any case). Because bias induction was unsuccessful, we could not analyze bias error as a separate error type. Therefore, we merged bias errors and other errors into one category in the main analyses.

### Main analyses

**Fig. 2:**
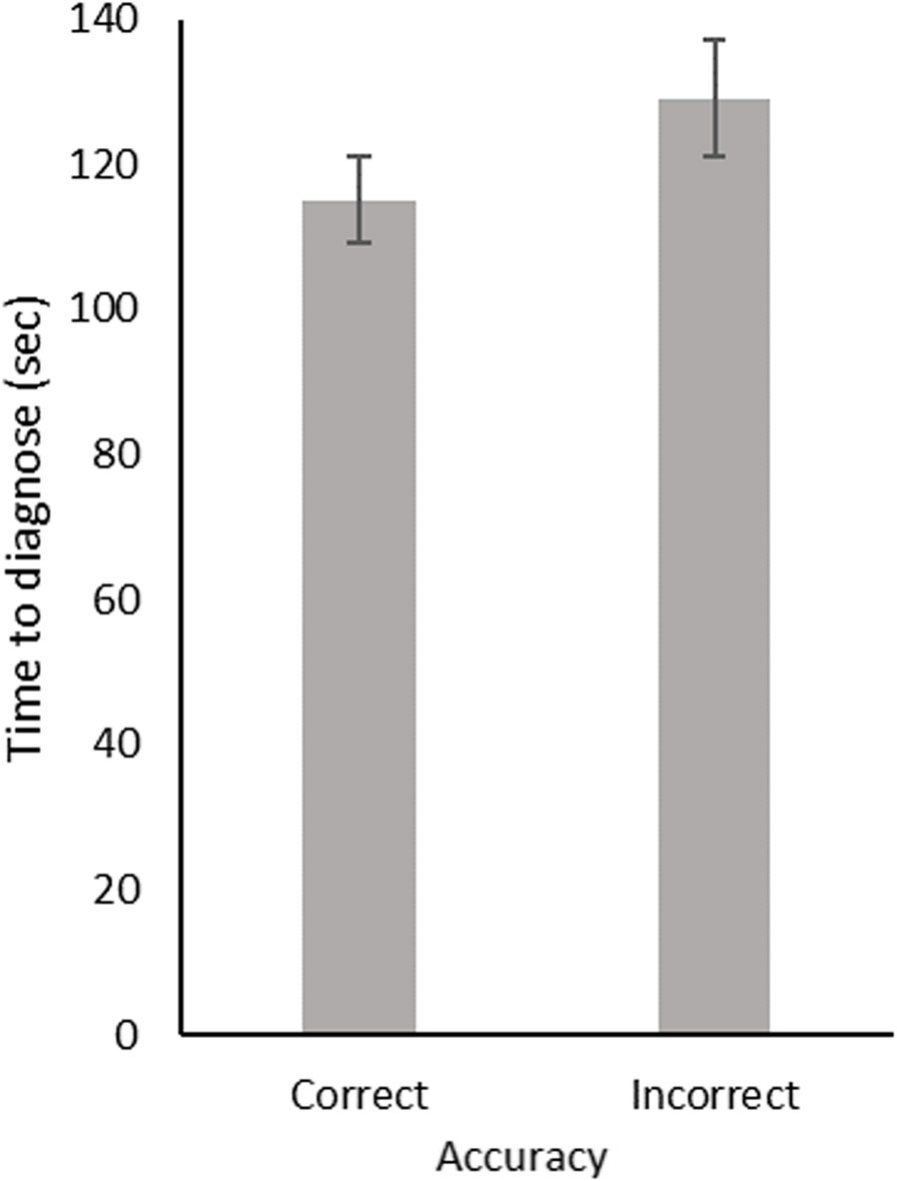
Mean time to diagnose (adjusted for case length) for correct and all incorrect diagnoses (*N* = 113). Bars indicate the 95% confidence interval

**Fig. 3:**
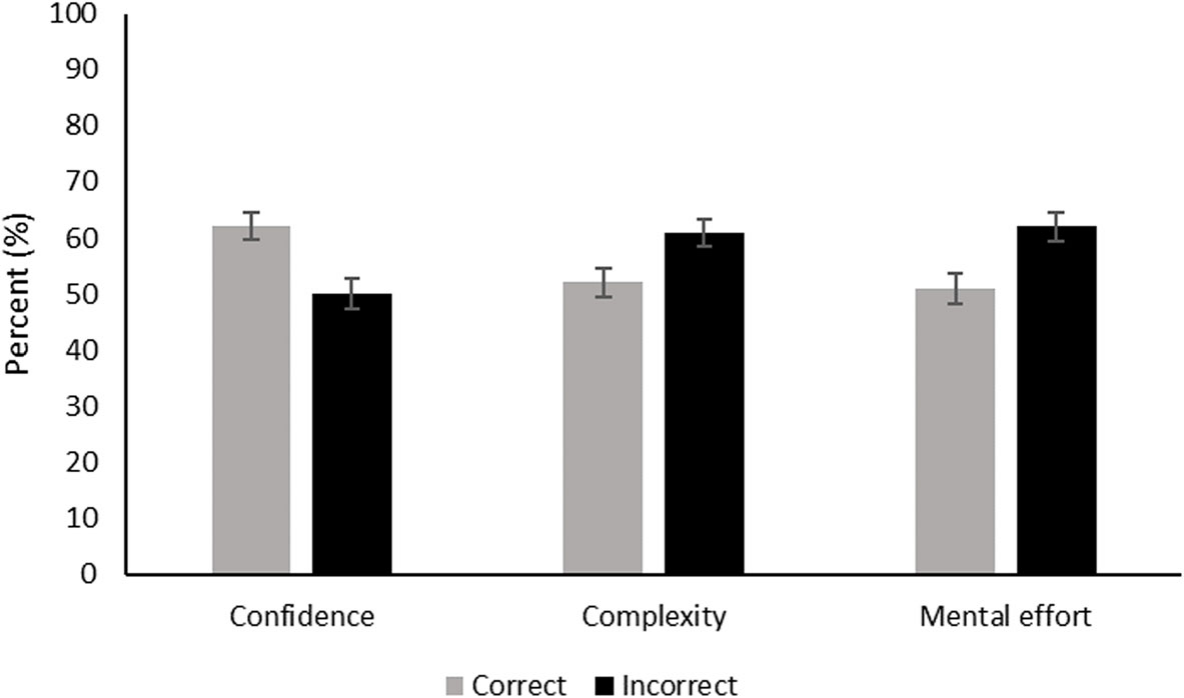
Mean confidence, complexity, and mental effort for correct and all incorrect diagnoses (*N* = 113). Bars indicate the 95% confidence interval

Residents were faster to reach a correct diagnosis than an incorrect diagnosis, *t* (112) = 4.51, *p* < .001, 95% CI [4.11 23.89], *d* = 0.37 (Fig. 2). Residents’ confidence was higher for correct diagnoses than for incorrect diagnoses, *t* (112) = 8.75, *p* < .001, 95% CI [8.48 15.52], *d* = 0.89 (Fig. 3).

### Exploratory analyses

#### Case complexity and mental effort

Residents found correct diagnoses less complex than incorrect diagnoses, *t* (113) = 7.51, *p* < .001, 95% CI [5.49 12.51], *d* = 0.67, and invested less effort in correct diagnoses as opposed to incorrect diagnoses, *t* (113) = 8.52, *p* < .001, 95% CI [7.23 14.77], *d* = 0.81 (Fig. 3).

#### Confidence-accuracy calibration

**Fig. 4:**
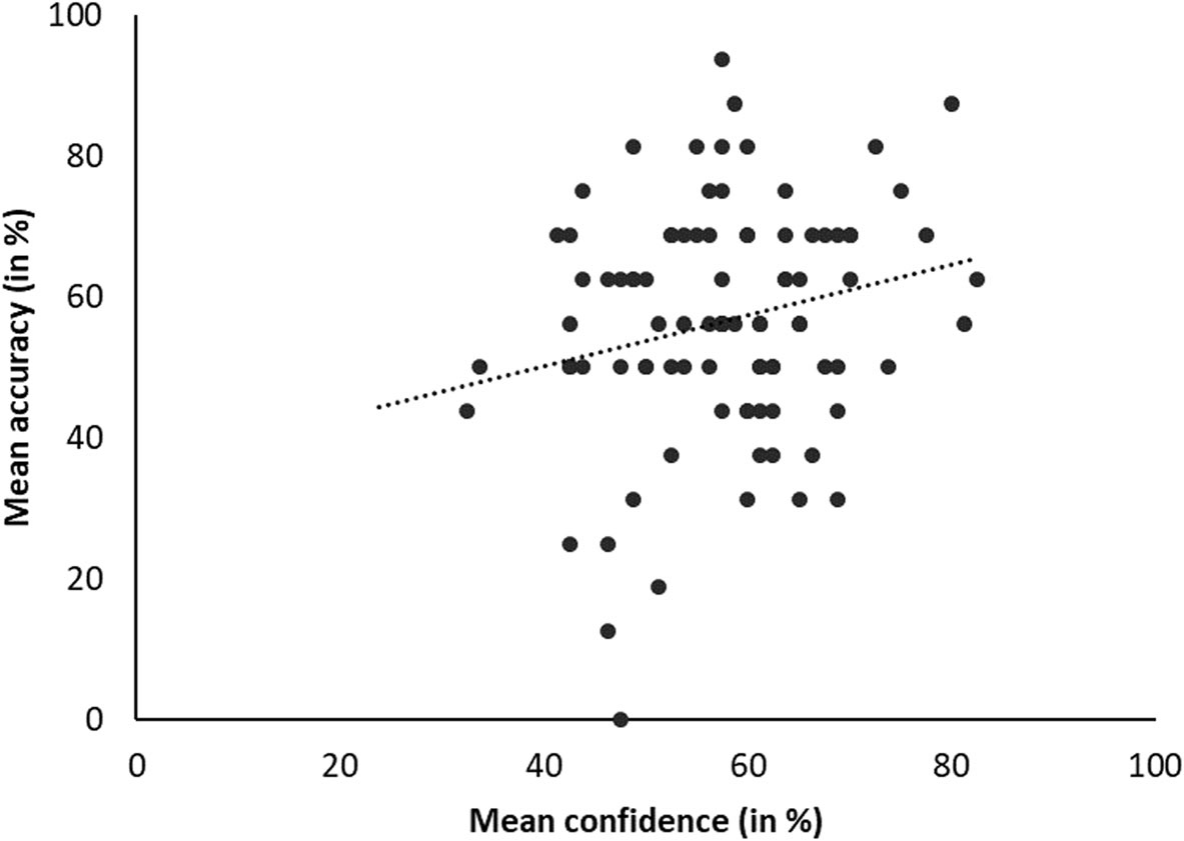
The relationship (linear trend line) between mean accuracy and mean confidence over all cases

Residents’ confidence-accuracy calibration trend line (Fig. 4) for average accuracy and confidence achieved a goodness-of-fit of *R*^*2*^ = 0.03, indicating that most residents were not well calibrated and that confidence-accuracy calibration varied widely between residents.

#### Resources

Residents indicated they wanted to consult one or more additional resources during diagnosis in 63% of the cases. We performed a repeated measures binomial logistic regression in RStudio, using the glmer package [30], to assess whether diagnostic accuracy was a predictor for resource use. We corrected for participant and case repetitions. The model showed no significant difference in how often residents indicated they wanted to use resources when they were correct (59%) versus when they were incorrect (68%), *b* = −.204, SE = 0.18, OR = 0.82, *p* > .05.

#### Bias diagnoses

Despite the overall unsuccessful bias induction, in several cases (opiate intoxication, hypoglycemia, tuberculosis, toxic megacolon) the bias diagnosis was given more frequently (although not significantly) on cases that were exposed to bias. Average time to diagnose and confidence (Table 2) were calculated in the same way as for correct diagnoses and other errors. We performed independent measures *t*-tests for these analyses, because the low numbers of bias responses would cause many data points to be excluded in a repeated measures test.

**Table 2:** Descriptive statistics for the time to diagnose (adjusted for case length) and confidence

*Error type*	*Time (sec)*			*Confidence (%)*		
	M	SD	95% CI	M	SD	95% CI
Correct (*n* = 96)	115	49.89	105–125	63	18.58	59–66
Bias (*n* = 28)	115	45.23	98–133	54	19.28	47–62
Other (*n* = 64)	136	54.85	122–150	47	20.37	42–52

Time to diagnose did not differ between bias errors and correct diagnoses, *t* (122) = − 0.03, *p* = .971, but a trend was present towards significance showing that bias errors were diagnosed faster than other errors, *t* (92) = 1.75, *p* = .082. Conversely, confidence showed a trend towards significance for residents to be less confident in bias errors than in correct diagnoses, *t* (122) = 2.07, *p* = .041, 95% CI [1.00 17.00], but no difference in confidence between bias errors and other errors, *t* (92) = 1.53, *p* = .130).

## Discussion

In this study we examined how time to diagnose related to diagnostic error. Because bias induction was unsuccessful we could not analyse bias errors and other errors separately. In line with our hypotheses, we found that even within subjects, residents took less time when they were correct and had more confidence in correct diagnoses. With this increased confidence, we also saw residents found correct cases were less complex and invested less effort in correct diagnoses. Additional analyses showed that residents’ confidence-accuracy calibration was poor and that accuracy did not influence how often residents requested resources. Further exploratory analyses of the bias errors were performed on the cases with a (non-significant) effect of bias. Although the results should be interpreted with caution, it was interesting that the results were in line with our hypotheses about correct diagnoses versus bias errors. Residents took equal amounts of time to diagnose correct and bias diagnoses (Table 2) and we saw a trend for bias errors to be reached faster than other errors. Contrary to our hypotheses, we found that confidence was similar between bias errors and other errors (Table 2) and that there was a trend for confidence to be lower for bias errors than for correct diagnoses.

Our findings regarding time to diagnose support and expand on the work of Norman et al. [23] and Sherbino et al. [22], who showed that physicians who diagnosed cases quickly were equally or more often correct than those who diagnosed cases more slowly. We have now shown that this applies on an individual level as well, i.e. physicians were faster when they were correct compared to incorrect, and that this cannot just be attributed to faster physicians being better diagnosticians. Further interesting insights come from the exploratory analyses where bias-induced errors showed a trend to be diagnosed faster than incorrect diagnoses (Table 2). These faster time to diagnose suggests that bias errors might differ from other types of errors.

This study and others show that fast diagnoses are not necessarily wrong and that correct diagnoses are not necessarily slow. The difference in time to diagnose between correct and incorrect diagnoses could partially be explained by the differences in relative difficulty of the cases: physicians could find some cases easier than other cases and might solve those cases quickly and correctly. The cases where they had more doubts would take longer. Although it is possible that this occurred in some cases, the fact that residents were poor judges of their performance, which was shown by their poor confidence-accuracy calibration and the small difference between use of resources for correct and incorrect diagnoses, speaks against this explanation for all cases taken together. This makes it unlikely that they consistently sped up or slowed down for cases where they were correct or incorrect. It is therefore less likely that time to diagnose for correct and incorrect cases can on average fully be explained by differences in case difficulty. Other causes of diagnostic errors need to be explored to gain better understanding of the diagnostic process. One such example would be knowledge deficits [13], which have also been shown to reduce cognitive biases [31].

Although our finding that correct diagnoses are made faster than incorrect diagnoses is not novel in itself, there is still a need to demonstrate and emphasize this finding: partially due to the pervasive notion that fast reasoning is primarily a cause for errors despite the findings of previous studies, and partially due to the limitations of these previous studies, which are in part overcome by the within-subjects design of the current study. Moreover, even though it seems logical that fast diagnosis is also a crucial part of the diagnostic process, many interventions focused on reducing errors in diagnostic reasoning still recommend stimulating analytical reasoning and slowing down the diagnostic reasoning process. Research that tested such interventions and educational strategies (such as the SLOW tool [32], general debiasing checklists [33] and cognitive forcing training [34]) did not show improved diagnostic accuracy [35]. Therefore, these interventions could result in harm because they would target both bias errors and correct diagnoses. It could be that reconsidering correctly diagnosed cases would result in more diagnostic tests and consequently overdiagnosis, which could also be harmful for patients [36].

The obvious solution would be to slow down only when necessary. However, this study confirms Meyer et al. [24]‘s finding that clinicians’ confidence is not well calibrated with accuracy and they do not ask for additional resources when necessary, whether they are residents or experts. Further, correct diagnoses and bias errors were similar, which makes it hard to differentiate between them. This suggests it would be difficult to use the concept of fast versus slow reasoning to detect diagnostic errors. Additional research is necessary to identify means to improve clinicians’ calibration, for example through feedback [37].

This study has several strengths and limitations. Strengths are that our study is a multi-center study with a randomized within-subjects design, which made the residents their own control and reduced variance between subjects. We additionally induced bias prospectively instead of assessing it retrospectively, which avoids issues like hindsight bias [38]. However, not all residents were vulnerable to bias and because we ended up with a small number of bias errors we were unable to replicate the induction of availability bias in Mamede et al.’s study [6]. This limited the analyses we could perform, because residents could only be biased to 4 cases at most, so the computed means for time to diagnose and confidence sometimes contain only one value for a resident, making the exploratory analyses less robust. However, we thought it best to be strict in our definition and selection of bias responses in order to approximate errors due to bias as closely as possible. It is unclear why bias induction was unsuccessful. One explanation is that the cases we developed to induce bias had many possible underlying diseases: this could have resulted in there being many possible differential diagnoses, which may have induced some analytical reasoning.

A further limitation is that our sample included a relatively large range of years of experience. It could be that the effects of time to diagnose and confidence are different for different levels of experience. This should be studied in a follow-up study. A final limitation is the use of written case vignettes: these limit the ecological validity of the study and do not allow residents to look up extra information while diagnosing the case. However, written cases provided the best way to prospectively induce bias and have been shown to offer a good approximation of real clinician performance [39, 40].

In conclusion, this study shows that correct diagnoses are reached faster than incorrect diagnoses and that this is not due to faster physicians being better diagnosticians. This indicates that fast diagnostic reasoning underlies correct diagnoses and does not necessarily lead to diagnostic errors. Exploratory analyses indicate that this might be different for diagnostic errors caused by cognitive biases, although more research into the characteristics of cognitive biases would be necessary to determine this. Both diagnostic error interventions and educational strategies should not promote focusing on slowing down to reduce errors and the common view of fast reasoning primarily being a cause for errors should be reconsidered.

## Supplementary Information

**Additional file 1:** Example of a clinical case.

**Additional file 2:** Survey questions.

## Data Availability

The dataset used and/or analysed during the current study are available from the corresponding author upon reasonable request. The study protocol was preregistered and is available online at Open Science Framework (https://osf.io/ga4ed/?view_only=e5e575fdd7b0452f92c668c0b3f0a363, DOI: 10.17605/OSF.IO/GA4ED).

## Abbreviations

ANOVA: Analysis of variance
ANCOVA: Analysis of covariance
DPT: Dual process theory
EBV: Epstein-Barr virus
NASEM: National Academies of Sciences, Engineering, and Medicine
TBC: Tuberculosis
