# Supplementary material for: The relationship between time to diagnose and diagnostic accuracy among internal medicine residents: a randomized experiment
Source: BMC Med Educ. 2021 Apr 21;21:227. doi: 10.1186/s12909-021-02671-2 (PMC8061054; doi:10.1186/s12909-021-02671-2)
Supplement: Supplementary file 1 — Additional file 1: Example of a clinical case. [file 12909_2021_2671_MOESM1_ESM.docx]

**Appendix A – Example of a clinical case**

Biasing case A

**History of present illness**

A 68-year old man is referred to the emergency room because of shortness of breath. He has had flu symptoms for two weeks. His wife has had the same symptoms, and she has since recovered well. The last two days he has started coughing up sputum. The sputum was green at first, but since this morning it has been rust brown. In addition, he suddenly became much sicker today. His wife thinks he is less alert than usual. Deep breathing causes pain in his left chest half. He does not smoke, and he drinks alcohol in moderation.

**Past medical history**

Hypertension – managed by PCP with Perindopril.

Hypercholesterolemia – treated with a statin.

**Physical examination**

Temperature 101.3°F. Oxygen saturation 94% (94-100%) on room air. Blood pressure 120/80 mmHg. Pulse 95 bpm. Moderately sick appearing man. JVD: not elevated.

Cardiac exam: normal auscultation without murmurs.

Lung exam: auscultation with crepitation in left posterior basal lung field.

The remainder of the physical examination was normal.

**Laboratory testing**

Leukocytes 12x109/L (<10 x 109/L), CRP 76 mg/L (<10 mg/L). Electrolytes and liver chemistry are within normal ranges.

**Additional testing**

CXR: good expiratory view. Left basal consolidation. No pleural effusion.

ECG: sinus rhythm.

**Diagnosis:** pneumonia.
