# Supplementary material for: The relationship between time to diagnose and diagnostic accuracy among internal medicine residents: a randomized experiment
Source: BMC Med Educ. 2021 Apr 21;21:227. doi: 10.1186/s12909-021-02671-2 (PMC8061054; doi:10.1186/s12909-021-02671-2)
Supplement: Supplementary file 2 — Additional file 2: Survey questions. [file 12909_2021_2671_MOESM2_ESM.docx]

**Appendix B – Survey questions**

Diagnosing cases:

First, the participants will be asked to evaluate 6 clinical cases. For each case the working diagnosis has been provided. For each case the participants is asked the following questions:

1. Indicate in percentages (%) the probability that the working diagnosis is correct:
2. Indicate how difficult it was to diagnose this case: (scale from 0-10, easy to difficult)

Then the participants will see 8 clinical cases without a working diagnosis. They are asked to diagnose each case themselves. For each case the participants will be asked the following questions:

1. What is the most likely diagnosis?
2. How confident are you in your diagnosis? (scale from 0-10, little confidence to a lot of confidence)
3. How difficult did you find it to diagnose the case? (scale from 0-10, easy to difficult)

Relevant personal information:

1. How old are you?
2. What is your gender?
3. Are you dyslexic?
4. In which year did you finish your study in Medicine?
5. How many years have you been a resident of internal medicine?
6. Which subspecialism do you want to, or will you, practice after residency?
7. How many years of experience do you have in clinical practice?
8. How many years of this experience did you acquire outside of your residency?

Feedback on the study:

1. What do you think the goal of this study is?

Finally, we ask the participants to provide some general feedback (if they have more to remark than what was asked in the previous questions).

1. Describe additional points that caught your attention during the study (e.g. mistakes in terminology, difficulty of the cases etc.) and, if necessary, elaborate on the answers provided on the previous feedback points.

The last optional question is whether participants would like to receive information about the study and its outcomes (and their own performance) when study 1 and 2 have concluded. If so, they can leave their email address.
